# Honey: A Therapeutic Agent for Disorders of the Skin

**DOI:** 10.5195/cajgh.2016.241

**Published:** 2016-08-04

**Authors:** Pauline McLoone, Afolabi Oluwadun, Mary Warnock, Lorna Fyfe

**Affiliations:** 1 Department of Biomedical Sciences, School of Medicine, Nazarbayev University, Astana, Kazakhstan;; 2 Department of Medical Microbiology and Parasitology Olabisi Onabanjo University, Sagamu, Ogun State, Nigeria;; 3 Dietetics, Nutrition and Biological Sciences, Queen Margaret University, Musselburgh, East Lothian, Scotland, United Kingdom

**Keywords:** dermatology, honey, skin cancer, wound healing

## Abstract

Problems with conventional treatments for a range of dermatological disorders have led scientists to search for new compounds of therapeutic value. Efforts have included the evaluation of natural products such as honey. Manuka honey, for example, has been scientifically recognised for its anti-microbial and wound healing properties and is now used clinically as a topical treatment for wound infections. In this review, scientific evidence for the effectiveness of honey in the treatment of wounds and other skin conditions is evaluated. A plethora of *in vitro* studies have revealed that honeys from all over the world have potent antimicrobial activity against skin relevant microbes. Moreover, a number of *in vitro* studies suggest that honey is able to modulate the skin immune system. Clinical research has shown honey to be efficacious in promoting the healing of partial thickness burn wounds while its effectiveness in the treatment of non-burn acute wounds and chronic wounds is conflicted. Published research investigating the efficacy of honey in the treatment of other types of skin disorders is limited. Nevertheless, positive effects have been reported, for example, kanuka honey from New Zealand was shown to have therapeutic value in the treatment of rosacea. Anti-carcinogenic effects of honey have also been observed *in vitro* and in a murine model of melanoma. It can be concluded that honey is a biologically active and clinically interesting substance but more research is necessary for a comprehensive understanding of its medicinal value in dermatology.

Historically, honey has been recognised around the world for its healing properties with records of its therapeutic use dating back to 2000 B.C. The ancient Greeks and Egyptians, for example, used honey to treat skin wounds and burns by applying topically on the skin.^1^ Honey has been reported to ameliorate a broad array of diseases but the focus of this review is on the therapeutic properties of honey in the treatment of disorders of the skin.

Traditional medicine in numerous countries around the world has described honey as efficacious in the treatment of a range of skin disorders. In Malaysian tradition, honey is used to treat furuncles, carbuncles, diabetic wounds and burns. Persian traditional medicine documented honey as effective in the treatment of wounds, eczema, and inflammation.^2^,^3^ In Ayurvedic medicine, a traditional medicine native to the Indian subcontinent, honey is used to treat cuts and wounds, eczema, dermatitis, burns, skin diseases and Fournier’s gangrene.^4^–^6^ Similarly, Quranic medicine in Pakistan recorded honey combined with cinnamon powder as a treatment for pustules, eczema, ringworm and a variety of other skin diseases and in Burkina Faso, Africa, it has been reported that indigenous people use honey as a skin cleansing agent and as a treatment for measles rash.^7^,^8^ The uses of honey in traditional medicine are still significant today, especially, when we consider the fact that most of the population of developing countries presently rely on indigenous medicine as their source of primary health care.^9^ Honey has also been used extensively as an ingredient in cosmetic skin care products both in the past and present day.^5^,^10^

In clinical practice today, manuka honey produced by honey bees (*Apis mellifera*) feeding on the manuka tree (*Leptospermum scoparium*) in New Zealand is used topically in the management of wound infections.^11^ It has been approved for clinical use in Australia, New Zealand, Europe, United States of America, Canada and Hong Kong and products include γ irradiated honey in gels, ointments and impregnated dressings. Revamil honey is another medical grade honey commonly used in clinical practice for wound care.^12^ It is produced by manufacturers in the Netherlands in collaboration with the University of Wageningen and the Academic Medical Centre, Amsterdam. The manufacturers have disclosed that the honey is produced in greenhouses but further details about the origin of the honey have not been revealed.

The skin healing ability of honey has been attributed to its antimicrobial properties, its ability to modulate the skin’s immune system and promote tissue repair.^13^,^14^ This review explores clinical and scientific research investigating the efficacy of honey in the treatment of wounds and a variety of other skin disorders. A principle aim was to use the scientific literature to evaluate the potential efficacy of honey in the treatment of a range of dermatological disorders.

## Methods

The databases Pubmed, Medline and ScienceDirect were used to carry out a comprehensive search of the scientific literature on the effects of honey in the treatment of skin disorders including wounds. Some of the key search terms used in combination were “honey” “antimicrobial activity” “skin immune system” “skin disorders” “wound healing” “seborrheic dermatitis” “atopic dermatitis” “psoriasis” “rosacea” “acne” “pityriasis versicolor” “cutaneous leishmaniasis” “skin cancer” “ Kazakhstan” and “Central Asia”. Relevant *in vitro* and *in vivo* studies were selected and we also searched the reference list of included papers to ensure that no important papers were omitted. Texts in English, published between 1990 and 2016 were included.

## Results

### The Efficacy of Honey in the Treatment of Skin Wounds

The ability of honey to aid the healing of skin wounds is the most widely researched aspect of honey as a therapeutic agent to date. A plethora of *in vitro* and *in vivo* studies have been performed.

#### The Efficacy of Honey in the Treatment of Skin Wounds: In Vitro Studies

*In vitro* studies have revealed that honey from diverse floral origins can kill a wide range of wound pathogens, including; methicillin resistant *Staphylococcus aureus* (MRSA), *Staphylococcus aureus*, *Escherichia coli*, *Pseudomonas aeruginosa* and *Acinetobacter baumannii.*^15^–^18^ As an example, the work of Cooper *et al*, (2002) demonstrated that manuka and pasture honey from New Zealand were active against 17 strains of *P. aeruginosa* isolated from infected burns with minimal inhibitory concentrations (MICs) below 10%.^19^ The authors concluded that these honeys have the potential to be effective treatments for burns infected with *P. aeruginosa.* Also, Cooper *et al* (2014) later demonstrated the ability of medihoney to disrupt the structure and inhibit the growth of *P. aeruginosa* biofilms grown *in vitro.*^20^ As well as killing microbes, studies have shown that sub-lethal concentrations of honey can reduce microbial pathogenicity, for example, Kronda *et al* (2013) demonstrated that sub-lethal concentrations of manuka honey reduced siderophore production, a virulence factor that scavenges iron for bacterial growth, in strains of *P. aeruginosa.*^21^ Even more remarkable are the *in vitro* findings that honey can reverse antimicrobial resistance. Jenkins and Cooper (2012) reported that manuka honey and oxacillin worked synergistically to inhibit the growth of MRSA and that manuka honey reversed oxacillin resistance in MRSA *in vitro*.^22^

Importantly, studies have also shown that honey from a variety of sources can modulate immunological parameters related to the skin immune system.^23^ For example, *in vitro*, honey has been shown to stimulate cytokine production by skin cells such as keratinocytes and other immune cells such as monocytes.^24^,^25^ It has been proposed that increased cytokine production in an early wound could enhance wound healing because cytokines such as TNF-α and IL-6 play an important role in the early wound healing process. Additionally, some studies have shown that honey or its extracts can down regulate the production of cellular molecules such as matrix metalloproteinases (MMPs) and reactive oxygen intermediates (ROIs) that may contribute to excessive inflammation in the chronic wound.^26^,^27^ It has been suggested that the immunomodulatory properties of honey may contribute to enhanced tissue repair or reduce chronic inflammation in the wound, leading to enhanced healing.

As well as its antimicrobial and immunomodulatory properties, honey has been shown to promote re-epithelialisation and angiogenesis in *in vitro* models of wound healing. Ranzato *et al* (2012) demonstrated that acacia, buckwheat and manuka honey, purchased at an apiculture centre in Okayamo, Japan, increased re-epithelialisation rates in scratch wounds induced in keratinocyte (HaCaT) monolayers.^28^ Furthermore, the mechanism was shown to be due to honey induced activation of pathways that regulate cell locomotion and cell proliferation. Barui *et al*, (2013) demonstrated that a honey alginate fibrous matrix induced faster re-epithelialisation than an alginate only matrix in a keratinocyte (HaCaT) wound model; E-cadherin protein was enhanced in the honey alginate model which may have promoted increased cell to cell adhesion.^29^ Rossiter *et al*, (2010) reported that the medicinal honey Activon containing 100% manuka honey, the honey based ointment Mesitran as well as a supermarket honey (Rowse) promoted angiogenic activity in a rat aortic ring assay *in vitro.*^30^

In conclusion, *in vitro* studies have revealed that honey has some remarkable scientific properties that, plausibly, could promote the healing of wounds.

#### The Efficacy of Honey in the Treatment of Skin Wounds: In Vivo Studies

Ideally, a wound will heal early but sometimes wound healing is delayed and this can be the result of systemic disease, malnutrition and infection of the wound leading to excessive inflammation. Indeed, excessive infiltration of neutrophils has been associated with deficient wound healing.^31^ Micro-organisms can sometimes attach to the wound bed and form a biofilm which is disruptive to the healing process. Wound infections exacerbate illness, cause anxiety and increase patient morbidity and mortality. Surgical wound infections lengthen hospital stay and chronic wounds require considerably more dressings. Hence, effective prevention and management of wound infections will impact positively on both patient health and cost.

There are a plethora of *in vivo* studies investigating the efficacy of honey in the treatment of wounds;^32^–^55^ many of the findings are controversial. However, a recent Cochrane based review by Jull *et al*, (2015)^56^ concluded that there is quality evidence that honey heals partial thickness burn wounds more quickly than conventional treatments and infected postoperative wounds more effectively than gauze or antiseptics. It was concluded that other studies comparing honey with conventional methods in wound healing were of insufficient quality to form any definitive conclusions.

Larger, well designed, double blind, clinical studies are required for a fuller understanding of the efficacy of honey in the treatment of different types of wounds. The mechanism of the skin healing properties of honey in relation to burn wounds is not fully understood but may, at least partially, be due to the antioxidant content of honey. There is evidence for free radical activity and reduced antioxidant scavenging capacity in burn wounds leading to oxidative stress.^57^ Honeys that are rich in antioxidants are likely to increase the antioxidant capacity of burn wounds and mop up free radicals leading to reduced oxidative stress.^58^ Of course, the antimicrobial and immunomodulatory properties of honey may also positively encourage the wound healing process in burn wounds.

Mode of administration and combination therapy with other agents such as antibiotics or other natural products could be considered. Techniques such as checkerboard and time kill studies are currently being used to determine the synergistic effects of antimicrobial agents (personal communication; Oluwadun A. & Akinduti P., Olabisi Onabanjo University).

### The Efficacy of Honey in the Treatment of Other Disorders of the Skin

#### In Vitro Studies

*In vitro* studies have revealed that honey can inhibit the growth of a range of dermatologically important microbes. As well as inhibition of microbes responsible for wound infections, honey has been shown to inhibit the growth of dermatophytes a cause of tinea infections, *Candida albicans* a cause of cutaneous candidiasis and *Propionibacterium acnes* a cause of acne.^16^,^59^–^61^ Many studies have demonstrated the antimicrobial effects of honey from a variety of sources against *S. aureus*. As well as wound infections *S. aureus* is an important cause of furuncles, styes and impetigo and super-infection with *S. aureus* is common in atopic dermatitis.^62^ Research should continue to investigate the *in vitro* effects of honey against other dermatologically important microbes such as *Malassezia* species, human papilloma virus and *Bacillus oleronius*.

Some skin disorders such as contact dermatitis, atopic dermatitis and psoriasis have been classified as immune mediated skin disorders. Although the aetiology of the majority of immune mediated skin disorders are not fully understood the immune system is believed to play a significant role in the pathogenesis of the disease. Such disorders commonly respond to treatment with immunomodulating agents such as corticosteroids or ultraviolet radiation therapy. Recently, *in vitro* studies have revealed that honey is able to modulate the immune system, for example, a study by Majtan *et al*, (2010) demonstrated that acacia honey from Slovakia stimulated TNF-α, TGF-β, IL-1β and matrix metalloproteinase 9 (MMP-9) mRNA expression by human primary keratinocytes isolated from human foreskin.^24^ Subsequently, Majtan *et al*, (2013) reported that an aqueous extract of fir honeydew honey from Slovakia inhibited TNF-α induced matrix metalloproteinase-9 (MMP-9) protein and mRNA production by human keratinocytes (HaCaT) cells.^27^ Since the role of the immune system in skin disorders is complex, it is difficult to infer what effects honey will have in the treatment of immune mediated skin disorders without further investigation. It is likely that both the origin of the honey and the microenvironment of the skin disorder will influence clinical outcome. Clearly, more research is necessary for a better understanding of the immunomodulatory properties of honey and their relevance for skin disease.

#### The Efficacy of Honey in the Treatment of Other Disorders of the Skin: In Vivo Studies

The majority of clinical studies performed to date have investigated the efficacy of honey in the treatment of skin wounds. There is a paucity of clinical studies investigating the effects of honey on other types of skin disorders; however, some of the studies that have been carried out have produced positive results. Al-Waili (2001) reported a remarkable improvement of symptoms in patients with seborrheic dermatitis (n=30) following topical application of a diluted crude honey (90%).^63^ The same researcher, later reported that a honey mixture containing natural honey of multi-floral origin from Lootah Farm, Al-Theed City, United Arab Emirates, olive oil and beeswax (1:1:1) markedly improved the symptoms of patients with atopic dermatitis (n=21) and psoriasis (n=18).^64^ Some of the psoriatic and atopic dermatitis patients received a honey mixture treatment in combination with corticosteroids and this allowed the concentration of corticosteroid to be reduced over time without exacerbation of symptoms. Al-Waili, (2003) suggested that the antimicrobial, anti-inflammatory and antioxidant properties of honey may explain the observed therapeutic effects. The same honey mixture was found to cure the symptoms of the fungal skin infections pityriasis versicolor in 79% of patients (n=14), tinea cruris in 71% of patients (n=14) and tinea corporis in 62% of patients (n=8).^65^ In 2005, Al-Waili reported that the same honey mixture significantly reduced mean lesion scores in infants with diaper dermatitis (n=12); the presence of *C. albicans* was found to be reduced in some patients treated with the honey mixture.^66^ In a small study (n=16) by Al-Waili (2004) it was reported that honey was more effective than acyclovir in the treatment of patients with labial and genital herpes simplex lesions, suggesting that honey could potentially be effective in the treatment of oral herpes simplex lesions.^67^

Acasia honey (Yamada bee farm, Japan) and the bee product Brazilian green propolis (BPE) have also been shown to be efficacious in the treatment of tinea infections *in vivo.^68^* Two hundred and forty two Congolese school children with either tinea capitis or pityriasis versicolor were treated with either 2% Miconazole (positive control), BPE (100mg/ml or 50mg/ml), acasia honey or Vaseline. The results showed that acasia honey, BPE at both concentrations and Miconazole significantly improved erythema, desquamation and pruritis in tinea patients in comparison to Vaseline.

Rosacea is an inflammatory skin disorder, characterised by facial redness, papules, pustules and telangiectasia. The bacterium *Bacillus oleronius* isolated from the *Dermodex folliculorum* mite has been implicated in the aetiology of the disease. A recent study by Braithwaite *et al* (2015) has shown that kanuka honey from New Zealand was efficacious in the treatment of rosacea.^69^ Their study included 138 participants with a diagnosis of rosacea and a Global Assessment of Rosacea Severity Score (IGA-RSS) of ≥ 2.69. Sixty nine participants were treated with topical Honevo (90% kanuka honey and 10% glycerine) for 8 weeks. The other 69 participants were treated with the control cream Cetomacrogol, a moisturising cream, commonly used as a vehicle for delivering topical medications. The results showed that 34.3% in the Honevo group and 17.4% in the control group had a ≥ 2 improvement in the IGA-RSS at week 8. The researchers concluded that Honevo is an effective treatment for rosacea and that future research should compare Honevo with other conventional treatments, such as topical Metronidazole and Azelaic cream, both of which have limited efficacy. The mechanisms of the therapeutic properties of kanuka honey in the treatment of rosacea are not fully understood but both the antibacterial and anti-inflammatory properties have been considered.

A recent study involving 136 participants with acne (Investigators Global Assessment (IGA) score of ≥ 2.68 aged between 16 and 40 years was carried out to investigate the efficacy of topical kanuka honey in the treatment of acne.^70^ Sixty eight of the participants were randomised to a treatment regime which involved applying Protex, a trilocarbon-based antibacterial soap twice daily for 12 weeks whilst the other 68 participants applied the anti-bacterial soap treatment followed by application of Honevo directly after washing off the bacterial soap, twice daily for 12 weeks. The results demonstrated that 4 out of 53 patients (7.6%) in the honey treated group and 1 out of 53 (1.9 %) patients in the anti-bacterial soap only treated group had a ≥ 2 improvement in IGA score. The authors concluded that there was no evidence that adding Honevo to standard anti-bacterial soap treatment for acne is more efficacious than anti-bacterial soap alone. The authors however did raise concerns about treatment compliance due to the young age of many of the participants and the high rate of withdrawal. Medical grade kanuka honey has also been tested for its efficacy in the treatment of eczema and psoriasis.^71^,^72^ No evidence of effectiveness in the treatment of eczema above that of an aqueous control cream was reported. The study involved 15 participants with bilateral eczematous lesions on the limbs; medical grade kanuka honey was applied to a representative lesion on one side and aqueous cream BP to the other, every night for 2 weeks. Aqueous cream is not a recommended treatment for eczema and therefore represented a negative control. The authors concluded that their study did not demonstrate any evidence that kanuka honey is an effective treatment for eczema, however, the small sample size and incomplete blinding were acknowledged as limitations of the study. The same study design was also used to investigate the efficacy of kanuka honey in the treatment of psoriasis. The results showed that kanuka honey was of similar efficacy to aqueous cream; a recommended treatment for psoriasis but with lower efficacy than corticosteroids. Medical grade kanuka honey has also been tested for its efficacy in the treatment of cold sores and compared with Acyclovir.^73^ The study showed that Kaplan-Meier estimates of median healing time were similar for honey and Acyclovir. However, limitations of the study were that participant size was small with only 15 patients; the authors proposed that a larger clinical study should be conducted.

Naidoo *et al* (2011) tested the efficacy of manuka honey as a prophylactic treatment for dermatitis in a phase II randomised controlled trial involving patients undergoing radiation therapy for breast cancer.^74^ 81 patients were enrolled in the study; 43 of which were treated with manuka honey and 38 with standard aqueous cream. The results showed that there was a lower incidence of grade >2 dermatitis in the patients treated with honey (37.2%) compared with those treated with aqueous cream (57.8%). When ≥ grade 2 dermatitis did occur the duration was shorter in the honey treated group in comparison to the group treated with aqueous cream.

One study also investigated the therapeutic value of honey in the treatment of cutaneous leishmaniasis.^75^ In this study, 90 patients with cutaneous leishmaniasis were allocated to a treatment regime; 45 patients were treated for 6 weeks with topical honey soaked gauze twice daily and intra-lesional injection of glucantime weekly. The other 45 patients were treated with intra-lesional injection of glucantime only. By the end of the treatment more patients had complete cure in the glucantime only treated group (71%) than in the glucantime and honey treated group (51.1%), suggesting that the honey used in this study is not of therapeutic value in the treatment of cutaneous leishmaniasis.

The aforementioned studies investigating the efficacy of honey in the treatment of other types of skin disorders are relatively small scale and several of them have been carried out by the same researcher, nevertheless, they support the possibility that honey may be therapeutic in the treatment of other types of skin disorder such as fungal skin infections and inflammatory skin conditions. Systematic reviews are important for evidence based method and this approach has been adopted by Jull *et al* (2015)^56^ to evaluate the efficacy of honey in the treatment of wounds. Clinical studies investigating the efficacy of honey in the treatment of other types of skin disorders are more limited and we have described all published findings irrespective of the quality of the study design. It is important that all future studies carried out follow international standards for clinical trial reporting. Undoubtedly, further research is necessary, incorporating *in vitro*, animal and clinical studies to determine the medical value of honey in the treatment of a range of dermatological disorders. Even if honey is found to be an ineffective treatment for certain skin diseases such studies are important because the knowledge will inform patients and clinicians considering alternative therapies for dermatological disorders.

### Honey and Skin Cancer

Recently, Fernandez-Cabezudo *et al*, (2013) reported that manuka honey could inhibit the proliferation and induce apoptosis in three cancer cell lines, one of which was the murine melanoma cell line B16.F1.^76^ Additionally, Pichichero *et al*, (2010) reported that acacia honey inhibited proliferation of murine and human melanoma cells by inducing cell cycle arrest at G_0_/G_1_.^77^ *In vivo*, a murine melanoma tumour model treated with intravenous manuka honey displayed a significant reduction in tumour growth.^76^ Some of the mice received co-administration of manuka honey and the chemotherapeutic drug taxol and this resulted in a significant inhibition of the growth of the tumour and improved overall animal survival suggesting that manuka honey, as well as having anti-tumourogenic properties, may reduce chemotherapy induced toxicity. No changes in haematological and chemical markers were observed in the mice treated with intravenous manuka honey suggesting that it is safe to administer honey in this way. In another study, tualang honey from Malaysia was shown to protect murine keratinocytes (PAM 212 cells) *in vitro* from the immunomodulatory and photocarcinogenic effects of UVB radiation.^78^ UVB irradiated keratinocytes treated with honey exhibited reduced expression of COX-2 and NF-κB activation in comparison to UVB only treated cells. Furthermore, UVB irradiated keratinocytes treated with tualang honey displayed a marked reduction in DNA damage in the form of cyclobutane pyrimidine dimers and 8-oxo-7, 8-dihydro-2-deoxyguanosine compared with UVB irradiated controls. Tualang honey may therefore be able to protect the skin against the immunomodulatory and photocarcinogenic effects of sunlight exposure.

The ability of honey to inhibit the proliferation of tumour cells is thought to be due to the various flavonoid and phenolic compounds present in honey. Evidence for this comes from the work of Pichichero *et al*, (2010; 2011) showing that chrysin, a flavanoid found in acacia honey inhibited proliferation of melanoma cells via cell cycle arrest and apoptosis.^77^,^79^ Honey has also been shown to regulate expression of p53, the tumour suppressor protein and down regulate Bcl-2 an anti-apoptotic protein, found at high levels in numerous cancers.^80^ The anti-inflammatory effects of honey may also contribute to its anti-carcinogenic properties, particularly as inflammation has been shown to contribute to the progression of cancer.^81^

The anti-carcinogenic properties of honey observed to date are promising but more research is necessary, particularly *in vivo*, for a fuller understanding of the potential efficacy of honey in the treatment or prevention of skin cancer.

## Discussion

The ability of honey *in vitro* to kill skin relevant microbes, alter microbial pathogenicity, reverse antibiotic resistance, modulate immunological parameters, promote tissue repair, inhibit tumour cell growth and protect against UV induced DNA damage is really quite remarkable considering it is a scientifically unaltered, purely natural substance produced by bees. *In vitro* studies have sparked excitement amongst researchers about the therapeutic potential of honey for clinical practice. Some of the properties observed *in vitro* are particularly relevant today when the current global crisis of antimicrobial drug resistance has rendered many infectious diseases, including wound infections, untreatable and malignant melanoma incidence is increasing faster than any other cancer.^82^,^83^ Skin Cancer is also a significant problem in Central Asian countries; in Kazakhstan for example, incidence figures from the Ministry of Health (2013) show that skin cancer is one of the most common forms of cancer along with lung and breast cancer.^84^ Furthermore, a particularly high incidence of basal cell carcinomas was recorded around the Semipalatinsk nuclear testing site in Kazakhstan.^85^

There are countless varieties of honey being produced worldwide and some may have superior healing abilities that are yet to be discovered. Central Asia possesses a unique biodiversity with open steppe, deserts and high mountains; honey production is abundant in Kazakhstan, Kyrgyzstan, Uzbekistan and Tajikistan. Beekeeping trailers enable honey producers to reach diverse locations in the region, despite this, the regions honeys have not been fully examined for potential biomedical uses. Other local bee products, such as propolis could also be investigated for their medicinal value as research has demonstrated antimicrobial, anti-carcinogenic and wound healing properties.^86^–^88^ The development of locally produced honeys into medical grade honeys suitable for use in clinical practice could be economically advantageous for the country concerned.

A recent review has highlighted that there is no statistical monitoring on the prevalence of chronic wounds in Kazakhstan and no approved protocols for wound care.^89^ The authors described that wound care products made from plant extracts have been developed in Kazakhstan although they have not been officially approved for clinical use. The review did not list honey as a treatment used for wound care in Kazakhstan despite its approved use in other parts of the world.

In conclusion, research has demonstrated that the bioactive properties of honey and the aetiology of skin diseases are complex and that there are considerable gaps in our knowledge and understanding of both. Innovative research that can maximally exploit the bioactive properties of this natural substance may in the future lead to the production of a medicinal product that is highly valued in dermatology.

## Figures and Tables

**Table 1:** Honey as a therapeutic agent for skin disorders; Summary of the key *in vitro* findings

**Key References**	**Key Findings (*in vitro)***
14–17,59–61,90,91	Honeys from around the world have potent antimicrobial activity against skin relevant microbes.
22	Honey can reverse antimicrobial resistance.
21,92–94	Pathogenicity of skin relevant microbes is reduced by honey.
23–25,95–99	Honey modulates cytokine production by cells of the skin immune system.
26,27,58,100	Anti-inflammatory effects of honey are observed *in vitro*.
28–30	Honey promotes re-epithelialisation and angiogenesis in *in vitro* wound models.
76–78	Honey induces apoptosis of a murine melanoma cell line and protects keratinocytes from the photocarcinogenic effects of UVB radiation.

**Table 2:** Honey as a therapeutic agent for skin disorders; Summary of the key *in vivo* findings

**Key References**	**Key Findings (*in vivo*)**
56	Clinical studies suggest that topical application of honey is more efficacious than conventional treatments in healing partial thickness burn wounds.
34,36–38,43,45,46,53	The efficacy of honey in the treatment of non-burn acute wounds and chronic wounds is controversial.
63–66,68,69	Limited human studies suggest that honey is therapeutic in the treatment of some inflammatory skin disorders and fungal skin infections.
76	Honey reduces tumour growth in a murine melanoma model.

**Table 3:** Studies investigating the efficacy of honey in the treatment of skin disorders (excluding wounds)

**Population**	**Honey Application**	**Outcome**	**Ref.**
37 patients; 14 with pityriasis versicolor, 8 with tinea corporis, 14 with tinea cruris and 1 with tinea faciei	Honey mixture containing honey, olive oil and beeswax (1:1:1) applied to the lesions 3 times daily for a maximum of 4 weeks. Honey was multi-floral from the United Arab Emirates.	Complete cure obtained in 79% of patients with pityriasis versicolor; 71% of patients with tinea cruris and 62% of patients with tinea corporis. Patient with tinea faciei obtained clinical cure 3 weeks after start of therapy.	65
242 Congolese school children with either tinea capitis or pityriasis versicolor	Treated with either 2% Miconazole, Brazilian green propolis extract or acasia honey (Yamada bee farm, Japan) or Vaseline.	Acasia honey (p < 0.05), Brazilian green propolis extract (p < 0.05) and 2% Miconazole (p < 0.01) significantly improved erythema, desquamation and pruritis in tinea patients in comparison to Vaseline.	68
10 patients with atopic dermatitis	Lesions on the right side of the body treated with vaseline. Lesions on the left side of the body treated with a multifloral honey mixture, containing honey beeswax and olive oil in a ratio of 1:1:1 for 2 weeks. Each treatment was applied three times daily. Honey was from the United Arab Emirates.	Significant improvement was seen in lesion scores on the left side of the body in 8 out of the 10 patients.	64
8 patients with psoriasis	Lesions on the right side of the body were treated with paraffin and lesions on the left were treated with honey mixture (as described above), 3 times daily for 3 weeks.	Significant improvement was seen in lesion scores on the left side of the body in 5 out of 8 patients.	64
12 infants with diaper dermatitis	Topical application 4 times daily with a multifloral honey mixture containing honey, beeswax and olive oil in a ratio of 1:1:1 for 7 days.	Mean total rash score at baseline was 2.91 ± 0.79. Decreased to 0.66 ± 0.98 at day 7. At the end of the study 10 of the 12 infants had either mild or no diaper dermatitis.	66
81 patients undergoing radiation therapy for breast cancer	Prophylatic treatment: 43 treated with a pure sterilized manuka honey UMF=18. Thirty eight patients treated with standard aqueous cream. Topical treatments were applied twice daily starting on day 1 of radiation and continued until 10 days post treatment.	Lower incidence of > grade 2 dermatitis in the patients treated with honey (37.2%) compared with those treated with aqueous cream (57.8%). When ≥ grade 2 dermatitis did occur duration was shorter in honey treated group. p = 0.08	74
138 patients with rosacea (IGA-RSS) ≥ 2.69	69 patients treated with topical application of Honevo (90% kanuka honey and 10% glycerine) for 8 weeks. 69 patients treated with the control cream Cetomacrogol.	34.3 % in the Honevo group and 17.4% in the control group had a ≥ 2 improvement in the IGA-RSS at week 8. p = 0.02	69
136 patients aged 16-40 years with acne IGA ≥ 2.68	68 participants applied Protex, a trilocarbon based antibacterial soap twice daily for 12 weeks. Another 68 participants followed the antibacterial soap treatment regime and applied Honevo (90% kanuka honey and 10% glycerine) directly after washing off the anti-bacterial soap, twice daily for 12 weeks.	4/53 (7.6%) of participants in the honey group and 1/53 (1.9%) in the control group had a ≥ 2 improvement in IGA score at week 12. Trial did not show evidence that adding Honevo to the antibacterial soap regime was more effective than soap alone.	70
15 patients with bilateral eczematous lesions on the limbs	Medical grade kanuka honey was applied to a representative lesion on one side and aqueous cream BP on the other, every night for 2 weeks.	Kanuka honey was not more efficacious than aqueous cream BP in the treatment of eczema. Aqueous cream BP is not a recommended treatment for eczema.	71
15 patients with psoriasis with bilateral lesions on the limbs.	Medical grade kanuka honey was applied to a representative lesion on one side and aqueous cream BP on the other, every night for 2 weeks.	Efficacy was similar to that of the aqueous cream which is a recommended treatment for psoriasis.	72
15 participants aged 16 or over with recurrent Herpes Simplex Labialis	Participants applied either medical grade kanuka honey or acyclovir to the lesion 5 times per day until the lesion resolved.	Kaplan-meier estimates of median healing time were similar for honey and acyclovir.	73
90 patients with cutaneous leishmaniasis	45 patients treated with topical honey twice daily along with intra-lesional injection of glucantime once weekly for a maximum of 6 weeks. 45 patients treated with glucantime only.	More patients had complete cure in the glucantime only treated group (71%) than in the glucantime and honey treated group (51%). p = 0.04	75
